# Prevalence of Neural Autoantibodies in Epilepsy of Unknown Etiology: Systematic Review and Meta-Analysis

**DOI:** 10.3390/brainsci11030392

**Published:** 2021-03-19

**Authors:** Pablo Cabezudo-García, Natalia Mena-Vázquez, Nicolás L. Ciano-Petersen, Guillermina García-Martín, Guillermo Estivill-Torrús, Pedro J. Serrano-Castro

**Affiliations:** 1 Instituto de Investigación Biomédica de Málaga-IBIMA, 29010 Málaga, Spain; lundahl151@gmail.com (N.L.C.-P.); guillerminagmartin@gmail.com (G.G.-M.); estivill.guillermo@gmail.com (G.E.-T.); pedro.serrano.c@gmail.com (P.J.S.-C.); 2 Unidad de Gestión Clínica de Neurociencias, Hospital Regional Universitario de Málaga, 29010 Málaga, Spain; 3 Unidad de Gestión Clínica de Reumatología, Hospital Regional Universitario de Málaga, 29010 Málaga, Spain

**Keywords:** epilepsy, autoimmune epilepsy, antibodies, autoantibodies, neural autoantibodies, prevalence

## Abstract

Background: The prevalence of neural autoantibodies in epilepsy of unknown etiology varies among studies. We aimed to conduct a systematic review and meta-analysis to determine the pooled global prevalence and the prevalence for each antibody. Methods: A systematic search was conducted for studies that included prospectively patients ≥16 years old with epilepsy of unknown etiology and systematically determined neural autoantibodies. A meta-analysis was undertaken to estimate pooled prevalence in total patients with a positive result for at least one neural autoantibody in serum and/or cerebrospinal fluid (CSF) and for each autoantibody. Results: Ten of the eleven studies that met the inclusion criteria and a total of 1302 patients with epilepsy of unknown etiology were included in themeta-analysis. The global pooled prevalence (IC95%) was 7.6% (4.6–11.2) in a total of 82 patients with a positive result for any neural autoantibody. None of the controls available in the studies had a positive result. Individual pooled prevalence for each autoantibody was: glycine receptor (GlyR) (3.2%), glutamic acid decarboxylase (GAD) (1.9%), *N*-methyl-d-aspartate receptor (NMDAR) (1.8%), leucine-rich glioma inactivated-1 protein (LGI1) (1.1%), contactin-2-associated protein (CASPR2) (0.6%) and onconeuronal (0.2%). Conclusions: The pooled prevalence of neural autoantibodies in patients with epilepsy of unknown etiology is small but not irrelevant. None of the controls had a positive result. There was high heterogeneity among studies. In the future, a homogeneous protocol for testing neural autoantibodies is recommended.

## 1. Introduction

Epileptic seizures are one of the most frequent clinical presentations of autoimmune encephalitis [1], and in some cases, it may be the only symptom [2]. Immune epilepsy was firstly included in the ILAE Seizure Classification in 2017 [3], defined as patients with evidence of immune-mediated inflammatory disease in the central nervous system. This evidence is based on MRI and cerebrospinal fluid (CSF) findings, clinical response to immunotherapy, and the presence of neural autoantibodies in serum or CSF [4].

In recent publications, many experts have considered that not all seizures triggered by immune damage to the brain should be considered as immune epilepsy [2] since epilepsy is understood as a chronic process [5]. With this in mind, they suggest two different concepts [6]: “acute symptomatic seizures secondary to autoimmune encephalitis” and “autoimmune-associated epilepsy”. The former consider cases in which immune encephalitis with positive neural surface antibodies presents with symptomatic seizures but reaches long-term seizure freedom with immune-targeted therapy [7]; the latter would account for those with antibodies targeting intracellular antigens such as glutamic acid decarboxylase (GAD) and onconeural protein antibodies, in which immunotherapy is frequently ineffective as a result of neural death and permanent brain damage [8]. In any case, being able to differentiate this in clinical practice is sometimes difficult, since some cases of autoimmune encephalitis associated with neural surface autoantibodies present as a “forme fruste” with drug-resistant seizures being their only manifestation, or at least the only apparent manifestation, being similar to the chronic course of “autoimmune-associated epilepsy” [6]. Also, patients harboring neural autoantibodies can present as new-onset epilepsy lacking other features of autoimmune encephalitis and reaching a favorable outcome without the need for immunotherapy [9].

Nevertheless, hypothetical autoimmune pathogenesis may exist in patients diagnosed with epilepsy of unknown etiology and could imply a new therapeutic approach with immune-targeted therapies, for example, in patients with drug-resistant epilepsy. Taking this into account, many studies have attempted to appraise the existence of different neural autoantibodies in series of patients with epilepsy of unknown etiology [10,11,12,13], others have also tried to characterize the features of those patients with positive antibodies [14,15,16,17], and even some of them have attempted to validate predictive models for neural-specific autoantibody status [18,19,20]. All those studies presented variable methodologies and results; therefore, we aim to conduct a systematic review of the literature and meta-analysis to identify those studies that performed a systematic approach for the detection of neural autoantibodies in patients with epilepsy of unknown etiology, and analyze its global prevalence as well as the individual prevalence of each autoantibody.

## 2. Materials and Methods

A systematic review was conducted of studies that related to the research question.

### 2.1. Study Identification and Selection

A systematic search was carried out in MEDLINE and Embase until 26 January 2021 using the following MeSH and free text terms: “epilepsy”, “seizure”, “autoantibody”, “autoantibodies”, “antibody”, and “antibodies”. It was restricted to the English language. In Embase, only articles were included (Supplementary Materials). The review protocol followed the declaration of Preferred Reporting Items for Systematic Reviews and Meta-Analyses (PRISMA) [21] and was registered in PROSPERO (CRD42020214838). A free secondary search was also conducted in MEDLINE database with the terms “epilepsy” and “autoantibody” or “antibody” and in the reference lists of selected articles. The search was performed by two researchers (P.C.-G. and N.M.-V.) who independently reviewed article titles and abstracts.

All types of studies were considered for inclusion except conference abstracts, case or cases series reports, and narrative reviews. Patients 16 years or older diagnosed with epilepsy according to the most recent ILAE definition (defined as at least two unprovoked seizures occurring >24 h apart orone unprovoked seizure and a probability of at least 60% of further seizures occurring over the next 10 years) [5] and unknown etiology (or cryptogenic) were included for the analysis. Patients with hippocampal sclerosis were not excluded in the absence of classical risk factors such as febrile seizures, central nervous systeminfection, and birth trauma. We excluded studies performed on pre-selected epilepsy sub-groups (except for adults, new-onset epilepsy, focal epilepsy, hippocampal sclerosis, and drug-resistant epilepsy). Studies that included patients with a prior diagnosis of encephalitis or studies including, exclusively, patients with suspicion of encephalitis before antibody testing were also excluded. Also, studies and patients with retrospective recruitment were excluded.

According to the type of intervention, inclusion criteria were as follows: studies in which systematic determination of neuronal autoantibodies in serum and/or CSF was carried out with a positive/negative result (following the method and criteria used in each of the studies). Studies and results regarding the determination of autoantibodies against uncharacterized antigens and GluR3 were excluded due to lack of specificity [22]. Detection of autoantibodies targeting uncharacterized antigens against the voltage-gated potassium channel-complex (VGKC complex) but without the presence of leucine-rich glioma inactivated-1 protein (LGI1) or contactin-2-associated protein (CASPR2) autoantibodies were excluded [23]. Regarding glutamic acid decarboxylase (GAD) autoantibodies, only high titters in serum/plasma or an intrathecal synthesis were considered positive. Serum/plasma titters of GAD antibodies considered high for each different method were: radioimmunoassay (RIA) > 1000 U/mL or 20 nmol/L; ELISA > 10000 IU/mL; immunoprecipitation assay (IPA) > 100 nmol/L; positive result in immunoblot; positive result in immunofluorescence (IF) using immunohistochemistry (IHC) or cell-based assay (CBA) [18,24,25]. Positive results for onconeuronal antibodies different from anti-Hu (anti-neuronal nuclear antibody, type 1 (ANNA-1), Ri (anti-neuronal nuclear antibody, type 2 (ANNA-2)), anti-neuronal nuclear antibody type 3 (ANNA-3), Ma-1/Ma-2, CV2 or anti-collapsing response-mediator protein-5 (CRMP5), amphiphysin, Purkinje cell cytoplasmic antibody, Type 2 (PCA2), and Purkinje cell cytoplasmic antibody, type Tr (PCA Tr), antibodies were not considered due to lack of clinically validated association with autoimmune epilepsy [4]. Positive results of control groups (if available) of the different studies were included to calculate risk difference.

The main outcome was global pooled prevalence, measured as the number (%) of patients with a positive result for at least one neural autoantibody in serum/plasma and/or CSF. The following neural autoantibodies were considered based on their reported association with autoimmune seizures and epilepsy and the frequency of its determination in the literature [4]: high titter GAD, onconeuronal antibodies, *N*-methyl-d-aspartate receptor (NMDAR), amino-3-hydroxy-5-methyl-4-isoxazolepropionic acid receptor (AMPAR) 1 and 2 (AMPAR1/AMPAR2), CASPR2, LGI1, gamma-aminobutyric acid (GABA) A and B receptor (GABAAR, GABABR), and glycine receptor (GlyR). Secondary outcomes were as follows: pooled prevalence of individual antibodies, recorded as the number (%) of patients with a positive result for a specific neural autoantibody.

### 2.2. Data Extraction and Bias Evaluation

The whole text was read in articles whose titles or abstracts met the inclusion criteria. The failure of any eligibility criterion was sufficient to exclude a study. Two reviewers (P.C.-G. and N.M.-V.) extracted the data from the documents, using an ad-hoc data sheet to record study variables (Supplementary Table S1). The level of the evidence was assessed using Scottish Intercollegiate Guidelines Network (SIGN) grading system [26]. Disagreement between reviewers on the inclusion/exclusion of studies was solved by consensus or with the assistance of a third reviewer (P.J.S.-C.).

### 2.3. Data Synthesis and Analysis

A random-effect model (restricted maximum likelihood) was chosen to estimate the global pooled prevalence rate, with a 95% confidence interval (CI) calculated as an effect measure for all outcomes. The risk differences were used to compared cases and controls. According to the Cochrane review guidelines [27], the pooled prevalence for individual antibodies by the random-effects model (DerSimonian and Laird method) was chosen if severe heterogeneity was present at I2 > 50%, otherwise, the fixed-effects model was adopted (Mantel–Haenszel method). The 95% CI, I2 as a measure for heterogeneity, and the Egger’s and Begg’s test as a measure for publication bias were calculated for each analyzed group.

## 3. Results

### 3.1. Search

**Figure 1:**
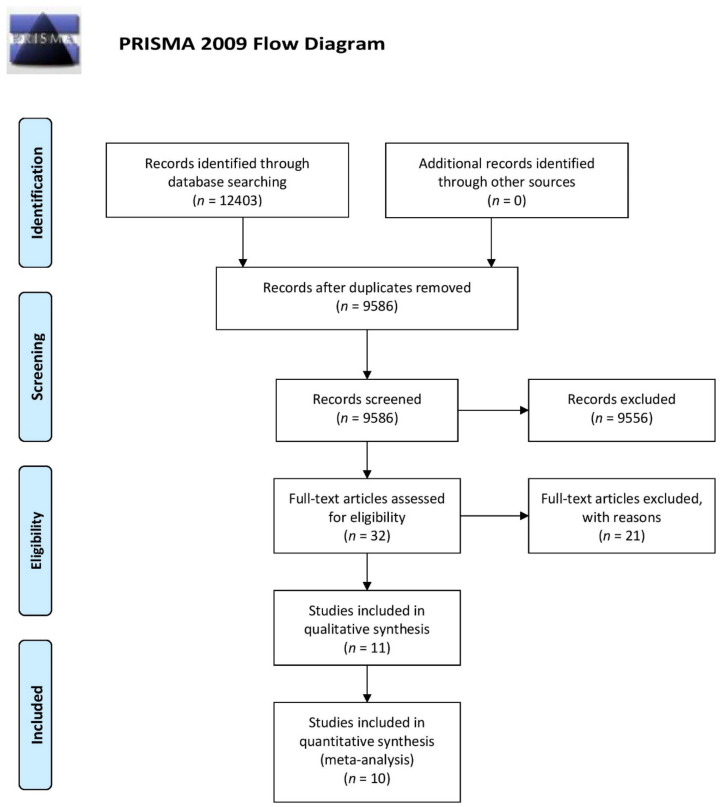
Preferred Reporting Items for Systematic Reviews and Meta-Analyses (PRISMA) flow diagram.

The literature searches initially retrieved 12,403 studies from which 2817 were excluded as duplicates, 9554 were excluded after reading the title and abstract, and 21 were excluded after reading the whole article (Supplementary Table S2). Therefore, 11 articles met the selection criteria (Figure 1). A total of 1361 patients with epilepsy of unknown etiology and at least one autoantibody tested in serum or CSF were included, and 503 controls were identified. Finally, 1302 patients from ten studies were included in the meta-analysis.

### 3.2. Characteristics of the Included Studies for Qualitative Synthesis

Regarding the geographic localization of the studies, five studies were conducted in European countries, four were conducted in Near East countries, and two were conducted in the USA. Six studies were performed exclusively in patients with focal epilepsy [10,15,16,17,19,20], with two of them conducted on patients with temporal lobe epilepsy [10,17]. Two studies were performed in cohorts of patients with drug-resistant epilepsy exclusively [14,20]. Also, the cohort in Iorio et al. [11] that was selected for our analysis consisted of only drug-resistant epilepsy patients.

All studies were cross-sectional except for the study by Iorio et al. [11], which had an observational prospective design. Only five studies had a control group, consisting of healthy subjects [11,13,15,16] and patients with neurological diseases other than epilepsy and autoimmune diseases [12]. The results of the control group in Iorio et al. [11] are not presented in the article.

None of the selected studies were performed exclusively in cohorts of patients with new-onset epilepsy or hippocampal sclerosis. All studies tested autoantibodies in serum, but none performed a systematic determination in CSF. In Bruijn et al. [19], Iorio et al. [11], and Falip et al. [17], CSF antibodies were determined only in some patients with a viable sample, and in Liimatainen et al. [13] CSF antibodies were determined only in patients with a positive result in serum. Anti-GAD was the most frequently tested autoantibody, and it was determined in all studies, mainly by RIA, but more recent studies used IF as screening instead, and then other methods for confirmation and titers. Neural surface autoantibodies were tested in eight studies. In these studies, the most frequently determined were NMDAR and LGI1, which were tested in all of the studies. There was variability in the frequency of determination of CASPR2, GABAB, AMPAR, and GlyR between the different studies. GABAA was only determined in one study. IF was used for the determination of this type of autoantibodies. In the included studies, two strategies were used: one consisting of screening by IF IHC and then confirmation by CBA, and the other by performing CBA from the beginning. Onconeural antibodies were tested in six studies, in half of them in two steps, first IF IHC and then confirmation by immunoblot/CBA, but in the rest, only CBA or immunoblot was performed (Table 1).

### 3.3. Neural Autoantibodies Pooled Prevalence

**Figure 2:**
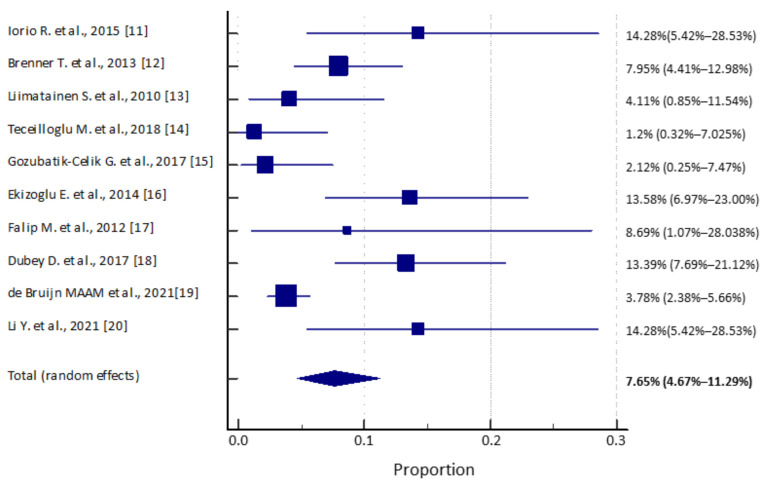
Pooled prevalence forest plot [11,12,13,14,15,16,17,18,19,20].

Ten studies were included for the global pooled prevalence meta-analysis after excluding Ansari et al. [9] by sensitivity analysis. The global pooled prevalence (IC95%) was 7.6% (4.6–11.2), with a total of 82 patients with epilepsy of unknown etiology with a positive result for any neural autoantibody (Figure 2). The prevalence in the different studies varied from 1.2% in the study of Tecellioglu et al. [14] to 14.2% in Li et al. [20] and Iorio et al. [11]. None of the 428 controls had a positive result.

Nine studies were included in the meta-analysis for individual pooled prevalence for each autoantibody. The study of Li et al. [20] was excluded, as it was not possible to individually extract the data from the prospective cohort (see Supplementary Table S1). In descending order, the prevalence for each autoantibody was as follows: GlyR (3.2%), GAD (1.9%), NMDAR (1.8%), LGI1 (1.0%), CASPR2 (0.6%), and onconeural antibodies (0.2%). Positive results for onconeuronal autoantibodies consisted of one patient for anti-Hu and another for Ma2/TA. In the studies with CSF available from some patients, only one patient harbored a positive result (NMDAR) in CSF but was negative in serum [19]. Table 2 shows the global and individual pooled prevalence with a 95% CI, as well as the number of studies and patients included in the analysis.

### 3.4. Heterogeneity and Publication Bias Assessment

The total cohort showed substantial heterogeneity (I 2 > 50%). The value of I2 to measure the heterogeneity of the global sample and for each antibody is shown in Table 2. The study of Ansari et al. [9] was excluded by sensitivity analysis. The Egger’s and Begg’s tests were used to evaluate publication bias in this meta-analysis. The bias was found to be statistically insignificant for the total cohort and for each antibody (Table 2).

## 4. Discussion

In this study, we attempted to estimate the pooled prevalence of neural autoantibodies in patients 16 years or older with epilepsy of unknown etiology through a systematic review of the literature and a meta-analysis. Our results show a pooled prevalence of 7.6% (IC95, 4.6–11.2%) in patients with epilepsy of unknown etiology. This suggests a possible immune-mediated mechanism in a low but not negligible proportion of patients with epilepsy of supposed unknown etiology and therefore the opportunity for a targeted treatment. It should be noted that this differs from the results of a different clinical scenario, which is the frequency of epileptic seizures in autoimmune encephalitis, where the prevalence is high [28].

The prevalence between studies varied from 1.2% in Tecellioglu et al. [14] and 14.28% in Iorio et al. [11] and Li et al. [20]. Interestingly, these three studies were performed in drug-resistant patients, a characteristic favoring a possible autoimmune origin [18,19]. The relatively low prevalence in Tecellioglu et al. was probable due to the methodology. In this study, the authors determined GAD65 by IRMA, being the only study included that used this method. We cannot say at the moment that immunoradiometric assay (IRMA) is less sensitive than RIA, but studies in other ambits found differences in the results of both methods [29]. Nevertheless, the comparatively low prevalence in this study can be explained because only testing for onconeuronals and anti-GAD and VGKC neural autoantibodies was performed. Some patients in this study were positive for VGKC but LGI1/CASPR2 were not tested, and hence we did not include them as a positive result. Determination of specific neural surface autoantibodies in this study could have increased the prevalence of positive results. Differences in prevalence were also found between the rest of the studies included in the meta-analysis, and the different methodologies and criteria for patient selection could also be a reason for this. For example, one of the studies with lower prevalence was the one from Bruijn et al. [19], where the included patients could not be suspicious of autoimmune etiology. In order to homogenize the sample for the analysis, we excluded studies with retrospective recruitment, where suspicion of autoimmune etiology is high.

In this systematic review, we also found high variability in the methodology for detecting neural autoantibodies among the included studies as well as which autoantibodies were considered for testing. We observed a great difference regarding the techniques used in the studies included to analyze GAD antibodies, and also in the titers that were considered high or very high (see Supplementary Table S1). Concerning neural surface autoantibodies, although the majority of the studies tested them by IF, some performed IHC first and then confirmed by CBA while others only performed CBA. In addition, the interpretation of what is a positive result by IF was variable between studies. For example, only some studies required confirmation by a second technique for certain autoantibodies [11,13,19,20], others excluded weak positive results and used higher dilution ratio in order to consider them positive [12,15,16], whereas Ansari et al. [10], which was excluded for meta-analysis by sensitivity analysis, considered weak positive results as positive, even with low dilution ratios, which could explain why it is the study with the highest pooled prevalence. In order to avoid pitfalls in the interpretation of neural antibodies results, it is advisable to perform panel testing of multiple autoantibodies rather than single antibody testing, screen both CSF and serum, and discuss cases with reference centers if antibodies directed to surface antigens are found at low titers in serum only, or if results do not match with the clinical presentation [30]. In recent studies in patients with epilepsy of suspected autoimmune etiology [31,32] where CSF analysis was systematically performed, only one patient had a positive result in CSF and was negative in serum [31]. Instead, eight patients had a positive result in serum but were not positive in CSF. Despite this, testing both CSF and serum in patients with suspected autoimmune encephalitis is essential [33], and hence should be also performed in clinical practice and future studiesof patients with epilepsy of suspected autoimmune etiology. Also, caution should be exercised when interpreting the results of commercial kits for onconeural antibodies because high frequency of false positive results and it is advisable to confirm positivity with at least two distinct techniques [34]. In future studies regarding neural autoantibody determination, it would be advisable to apply standardized methods [24,35,36] in order to achieve more homogenous results between studies.

None of the controls presented a positive result, regardless of whether some had other immune or neurological diseases. Despite most of the studies not having a control group, the above suggests that neural autoantibodies may play a role in the epilepsy of these patients. However, neural autoantibodies can also be found, albeit in a smaller proportions, in patients with epilepsy of other defined etiologies [12]. Additionally, some seropositive patients with epilepsy achieve a long-term outcome without the need of immunotherapy [9,37], so the mere presence of neural autoantibodies would not be enough to confirm a direct immune-mediated mechanism.

Regarding the pooled prevalence for each individual neural autoantibody, those with the highest pooled prevalence were GlyR, followed by GAD, NMDAR, and LGI1, with a high heterogeneity between studies. The prevalence for CASPR2 and onconeuronal autoantibodies was homogenous but with a lower frequency. Only one patient had a positive result for AMPAR, and none had a positive result for GABAB, suggesting that determining these antibodies in patients with epilepsy without other data suggestive of encephalitis is improbable, and that if detected, it is unlikely to have relevant significance. Instead, in view of the results, GlyR antibodies should be taken into account in future studies regarding immune epilepsies. These antibodies have been associated exclusively with long-term seizures without any other neurological symptom [38], with a better response to antiepileptic drugs, compared with other immune-based epilepsies [39], and with a response to immunotherapy in drug-resistant cases [40]. However, the high heterogeneity of their prevalence between studies makes it advisable to confirm results in future studies, with the drawback that testing for these antibodies can only be performed by a few laboratories. After GlyR, GAD was the most prevalent autoantibody in our analysis. This is not strange since, contrary to most neural autoantibodies, GAD antibodies have been associated for many years with drug-resistant epilepsies without meeting criteria of limbic encephalitis [4,6,25], which is congruent with the selection criteria of our study. The relatively high prevalence of NMDAR antibodies among other neural surface autoantibodies could be explained by the fact that NMDAR encephalitis is the most frequent autoimmune encephalitis [41]. Epileptic seizures can be the first symptom of many in NMDAR encephalitis [42], and in a few patients, it may be its only clinical expression [41,42]. However, in addition to the heterogeneity in the results, it should be taken into account that false positives and negatives of this antibody are not uncommon when performed in serum [43]. Our data also show that 1% of patients with epilepsy of unknown etiology can suffer from LGI1 encephalitis. Although this entity usually courses with typical features [44], with some even considered pathognomonic [45], epilepsy can be the presenting symptom, and memory and behavior alterations may be subtle or can be attributed to other causes [19]. Related to the previous point, is important to underlie that cognitive impairment is one of the classic manifestations of limbic encephalitis and sometimes is the predominant symptom (autoantibody-associated cognitive impairment) [46]. In patients with epilepsy of unknown etiology, a systematic cognitive exam revealing not only impaired working memory or short-term memory but also long-term memory formation may give us reason to suspect that there is an underlying autoimmune mechanism [47]. This could compensate for the lack of sensitivity in detecting subtle autoimmune encephalitis with actual criteria [33].

Our study presents some limitations. First, as occurs in many meta-analyses, there is heterogeneity between the results of the included studies due to small sample sizes and the variability in methodology and patient selection. To homogenize patients, we excluded studies with retrospective recruitment, studies performed exclusively in patients with high suspicion of autoimmune etiology, prior testing, or those in pre-selected epilepsy cohorts. An exception to the latter was the studies performed in drug-resistant epilepsy, hippocampal sclerosis, or new-onset epilepsy. The reason for this is that although these characteristics have been associated with autoimmune etiology, they are not specific if isolated [9,19]. These patients may be among those who benefit the most in routine clinical practice from the detection of a possible autoimmune mechanism. Second, in some studies the complementary exams to achieve the diagnosis of epilepsy of unknown etiology (i.e., high resolution MRI or long-term video-EEG) are not specified, and in others 1.5 T scanner was the exam performed, so it is possible that some patients included actually had a subtle structural or an idiopathic etiology. Nevertheless, we revisited with detail all papers and we excluded studies were the diagnosis of epilepsy of unknown etiology was doubtful (see Supplementary Table S2). Third, very few studies had control groups. Finally, the possibility of some of these patients having antibodies not yet unidentified should be considered.

In conclusion, the pooled prevalence of neural autoantibodies in patients with epilepsy of unknown etiology is small but not irrelevant. Even though the presence of these autoantibodies does not strictly imply an immune-mediated mechanism in all cases, the absence of these autoantibodies in controls favor this possibility and therefore the chance of a targeted treatment for some patients. The prevalence for each antibody obtained in our review and meta-analysis can guide research in terms of which autoantibodies may be determined in future studies as well as on clinical grounds. Heterogeneity in the type of antibody and in the method for its determination is high among studies. Further studies with homogeneous neural autoantibody testing protocols are needed in order to get a more accurate result of the actual prevalence of neural autoantibodies and also to find other parameters in order to improve the current understanding of the clinical significance of neuronal autoantibodies in patients with epilepsy of unknown origin.

## Figures and Tables

**Table 1:** Number of patients and antibody determination method of the included studies. AMPAR: amino-3-hydroxy-5-methyl-4-isoxazolepropionic acid receptor; CASPR2: contactin-2-associated protein; CBA: cell-based assay; GAD: glutamic acid decarboxylase; GlyR: glycine receptor; IHC: Immunohistochemistry; IRMA: immunoradiometric assay; LGI1: leucine-rich glioma inactivated-1 protein; NMDAR: *N*-methyl-d-aspartate receptor; RIA: radioimmunoassay. * not included in the meta-analysis.

Reference	*n* Patients Included for Meta-Analysis	Anti-GAD	Neural Surface Autoantibodies	Onconeuronal	Level of Evidence
Ansari B. et al., 2019 [10] *	*N* = 33 Control group: No	IF (CBA)	IF (CBA): NDMAR, AMPAR, LGI1, CASPR2, GABA_B_, GlyR	IF (CBA)	2−
Iorio R. et al., 2015 [11]	*N* = 42 Control group: *N* = 75	RIA	IF (IHC). Confirmed by CBA NMDAR, AMPAR, LGI1, CASPR2, GABA_B_,	IF (IHC). Confirmed by immunoblot	2+
Brenner T. et al., 2013 [12]	*N* = 176 Control group: *N* = 148	RIA	IF (CBA) NDMAR, LGI1, CASPR2, GLYR	-	2+
Liimatainen S. et al., 2010 [13]	*N* = 73 Control group: *N* = 200	RIA. Confirmation by IF/immunoblot	-	-	2++
Teceilloglu M. et al., 2018 [14]	*N* = 77 Control group: No	IRMA	-	Immunoblot	2+
Gozubatik-Celik G. et al., 2017 [15]	*N* = 94 Control group: *N* = 50	RIA	IF (CBA): NMDAR, AMPAR, LGI1, CASPR2, GABA_B_,	-	2+
Ekizoglu E. et al., 2014 [16]	*N* = 81 Control group: *N* = 30	IPA	IF (CBA): NMDAR, AMPAR, LGI1, CASPR2, GlyR	-	2++
Falip M. et al., 2012 [17]	*N* = 23 Control group: No	IF (IHC) and RIA	-	-	2+
Dubey D. et al., 2017 [18]	*N* = 112 Control group: No	RIA	Method not specified: NMDAR, AMPAR, LGI1, GABA_B_	Method not specified	2+
de Bruijn MAAM et al., 2021 [19]	*N* = 582 Control group: No	IF (IHC). Confirmation by CBA and ELISA	IF (IHC). Confirmation and GlyR by CBA NMDAR, AMPAR, LGI1, CASPR2, GABA_B_, GABA_A_, GlyR	IF (IHC). Confirmation by immunoblot	2++
Li Y. et al., 2021 [20]	*N* = 42 Control group: No	IF (IHC or CBA). Confirmation by other techniques	IF (IHC or CBA). Confirmation by other techniques NMDAR, AMPAR, LGI1, CASPR2, GABA_B_	IF (IHC or CBA). Confirmation by other techniques	2+

**Table 2:** The estimated pooled prevalence, the 95% confidence interval (CI), number of included studies and subjects, I2 as a measure for heterogeneity, *p*-value of the Egger’s test, and percentage of studies with low risk of bias for all meta-analyses.

Neural Autoantibody	*n*, Studies Determined	*n*, Subjects Determined	Pooled Prevalence	CI Lower	CI Upper	I-Squared	*p*-Value Egger’s Test	*p*-Value Begg’s Test
Total	11	1302	7.6%	4.6%	11.2%	75%	0.11	0.58
GlyR	3	839	3.2%	0.1%	9.8%	91%	0.20	0.60
GAD	9	1260	1.9%	0.6%	3.8%	70%	0.76	0.14
NMDAR	7	1129	1.8%	0.6%	3.7%	66%	0.08	0.75
LGI1	6	1087	1.0%	0.2%	2.7%	68%	0.36	0.09
CASPR2	6	1017	0.6%	0.2%	1.3%	47%	0.65	0.22
Onconeuronal	5	855	0.2%	0.1%	0.8%	38%	0.10	0.78

## Supplementary Materials

The following are available online at https://www.mdpi.com/2076-3425/11/3/392/s1: Supplementary Table S1: Data extraction sheet; Supplementary Table S2: Excluded studies and their reason.
