# Supplementary material for: Prevalence of Neural Autoantibodies in Epilepsy of Unknown Etiology: Systematic Review and Meta-Analysis
Source: Brain Sci. 2021 Mar 19;11(3):392. doi: 10.3390/brainsci11030392 (PMC8003737; doi:10.3390/brainsci11030392)
Supplement: Supplementary file 1 [file brainsci-11-00392-s001.pdf]

Supplementary Table S1: Data extraction sheet

REFERENCE	STUDY	PATIENTS	INTERVENTION	RESULTS	COMMENTS	LEVEL OF EVIDENCE (SIGN)
Li Y, 2021	Design:<br>Observational cross-sectional study<br><br>Objective:<br>To study the prevalence of serum Abs in patients ≥18 years of age with DRE of unknown cause before surgery and to propose and calculate a clinical APES (Antibody Prevalence in Epilepsy before Surgery) score for each subject<br><br>Recruitment:<br>Prospective and retrospective (two different groups)	Age: 44.84 ± 14.86<br><br>Focal DRE with no clear cause before surgery (a) ≥18 years of age; (b) diagnosis of focal DRE, who failed at least two anti-seizure medications; and (c) unknown cause of epilepsy. Exclusion criteria included the following: (a) generalized epilepsy; (b) definitive immune-mediated (autoimmune or paraneoplastic) epilepsy; (c) epilepsy with known cause, including but not limited to stroke, tumor, cortical dysplasia, epileptic syndrome such as tuberous sclerosis, and epilepsy with confirmed genetic etiology; and (d) pregnant. Brain MRI scan showing mesial temporal sclerosis (MTS) was not an exclusion criteria.<br><br>Number of patients included for metanalysis (Inclusion/Exclusion criteria met for our study):<br><br>N= 42.<br><br>Control group:	Serum<br>IF (ICH OR CBA); confirmed by other techniques)<br>- Onconeuronal<br>- NMDAR<br>- AMPA<br>- GABAb<br>- GAD65<br>- LGI1<br>- CASPR2	-Nº of total patients with positive result=6 (14.28%)<br><br>By autoantibody type:<br>- GAD65= 3*<br>- LGI1= 4*	- *One positive patient from retrospective cohort. Cannot extract specific results of % GAD65 and LGI1 for patients included in the study<br><br>-High titers considered for GAD65-Ab >20nmol/L	2+

		No				
Bruijn MAAM, 2021	Design:<br>Observational cross-sectional study<br><br>Objective:<br>to identify neuronal antibodies in a comprehensive cohort of patients with focal epilepsy of unknown etiology, and without, or with unrecognized, signs of encephalitis and to create a score to preselect patients requiring testing<br><br>Recruitment:<br>prospective, multicentre study	Age 18-89<br><br>adults with focal epilepsy of unknown etiology. (1) to include patients with epilepsy with or without additional symptoms, but with- out suspicion of encephalitis; and (2) to exclude patients strongly suspected of having AIE. Patients with epilepsy with known infectious, genetic, or metabolic etiologies were excluded. Patients with a structural lesion on brain magnetic resonance imaging (MRI) at inclusion were excluded, whereas patients with mesial temporal sclerosis, or with T2/fluid-attenuated inversion recovery (FLAIR) hyperintensities mainly in the mesial temporal lobe, both unilateral and bilateral, were not,<br><br>Number of patients included for metanalysis (Inclusion/Exclusion criteria met for our study):<br>N= 582<br>66 patients (11%) had an epilepsy duration of <1 year<br><br>Control group:	Serum (CSF If aviable, n=46)<br>All screened by IF ICH (except GlyR). If positive confirmation with other technics (CBA. GAD-65 also ELISA for titles; onconeuronals with immunoblot)<br>- Onconeuronals<br>- NMDAR<br>- AMPA<br>- GABAb<br>- GABAa<br>- GAD65<br>- LGI1<br>- CASPR2<br>- anti-GlyR	-Nº of total patients with positive result=22 (3,78%)<br><br>By autoantibody type:<br>- GAD65= 13 (2,2%)<br>- LGI1= 3 (0,5%)<br>- CASPR2= 3 (0,5%)<br>- NMDAR=1 (CSF) 0,17%<br>- anti-GlyR=2 (0,3%)	A subgroup of patients considered for epilepsy surgery were side by side tested by CBA, ELISA, and RIA for antibodies, as a part of a standardized protocol. In addition, patients with an ACES score of 2 or more were tested by commercial CBA and ELISA post hoc<br><br>GAD65 ELISA > 10000 IU/mL	2++

		No				
Ansari B,<br>2019	Design:<br>Observational cross-sectional study<br><br>Objective:<br>To investigate the difference in the prevalence of neural autoantibodies in patients with temporal lobe epilepsy of unknown etiology with hippocampal sclerosis vs without hippocampal sclerosis<br><br>Recruitment:<br>Prospective	Age 15-50<br><br>adult patients aged 15-50 years, diagnosed with International League Against Epilepsy (ILAE) and with TLE seizures. The patients with an obvious remote origin such as brain tumor, trauma, central nervous system (CNS) infection, vascular malformation, and generalized epilepsy were excluded.<br><br>Number of patients included for metanalysis (Inclusion/Exclusion criteria met for our study):<br><br>N=33 (17 with HS and 12 without HS)<br><br>Control group:<br>No	Serum IF CBA<br>-GAD-Ab<br>-Onconeuronal<br>-NMDAR-Ab<br>-AMPAR1/2-Ab<br>-LGI1-Ab<br>-CASPR2-Ab<br>-GABA B<br>-GLYR	-Nº of total patients with positive result=14 (42,4%). With HS n=6 (35,2%)<br><br>By Autoantibody type:<br>-GABA B= 11 (33,3%)<br>-NMDAR-Ab= 2 (6,0%)<br>-Onconeuronal= 1 (3,0%) [Tr+CV2 1]	- All positive results were weak positive.<br><br>- No difference HS vs non-HS<br><br>No included for metanalysis due to extreme value (also high frequency of GABAb no reproducible in other studies)	2-
Tecellioglu M,<br>2018	Design:<br>Observational cross-sectional study<br><br>Objective:<br>To determine the autoimmune and oncological antibodies in Adult drug-resistant epilepsy of unknown cause and identify the clinical, radiological, and EEG findings associated	patients with drug- resistant epilepsy of unknown cause. None of the patients included in the study had any neurological signs or neurological diseases other than epilepsy. Patients with focal and diffuse atrophy, nonspecific white matter lesions and idiopathic mesial temporal sclerosis (MTS) were not excluded. The seizures and syndromes were diagnosed according to the International League Against Epilepsy (ILAE) Commission on Classification	Plasma:<br>-Anti-GAD (IRMA) - Onconeuronal (Immunoblot, Euroinmun)	-Nº of patients with positive result= 1 (1,2%)<br><br>Bi autoantibody type:<br>-Onconeuronal= 1 anti-MA2/TA 1 patient)		2+

	with these antibodies according to data in the literature.<br><br>Recruitment:<br>Prospective 12 months	and Terminology 2017 [14]. The exclusion criteria were as follows:<br>1. Structural brain lesions (ischaemia, tumour, head trauma, vascular malformation, abscess, congenital malformation, heterotypic conditions).<br>2. Metabolic abnormalities (severe hypoglycaemia or hyperglycaemia, severe renal or hepatic deficiency, malignant hypertension, alcoholism).<br>3. Proven or suspected chromosomal anomalies and genetic syndromes.<br>4. Any malignancy<br><br>Number of patients included for metanalysis (Inclusion/Exclusion criteria met for our study):<br>N= 77 (HS not excluded)<br>> 18 years.<br><br>Control group:<br>No				
Dubey D, 2017	Design:<br>Observational cross-sectional study<br><br>Objective:<br>To determine the prevalence of neurological autoantibodies (Abs) among Adult patients	Age: 17-80 years<br><br>Patients with new- onset epilepsy or a history of epilepsy of unknown etiology. Patients were excluded they had underlying metabolic abnormalities that would explain their seizures (ie, severe renal or	Serum:<br>Method not specified<br><br>-NMDAR-Ab<br>-LGI1-Ab<br>-GABAb-Ab<br>-AMPAR-Ab<br>-Onconeuronal<br>- GAD65-Ab (RIA)	-Nº of patients with positive result=15 (13,39%)<br><br>Bi autoantibody type:<br>- NMDAR-Ab= 4 (3,57%)<br>-LGI1-Ab=4	-High titers considered for GAD65-Ab >20nmol/L	2+

	with epilepsy of unknown etiology<br><br>Recruitment: Prospective 12 months	hepatic failure, alcoholism, malignant hypertension, and severe hypoglycemia or hyperglycemia) or presence of structural brain lesions (eg, stroke, tumor, trauma, heterotopias, vascular malformation, abscess or infectious lesion, congenital malformations). Patients with idiopathic mesial temporal sclerosis were not excluded. suspected chromosomal anomaly, genetic syndromes, or in- born errors of metabolism leading to a syndrome of developmental delay with associated seizures were also excluded from the study.<br><br>Number of patients included for metanalysis (Inclusion/Exclusion criteria met for our study):<br>N=112 (HS not excluded)<br><br>Control group:<br>No		(3,57%)<br>-GAD65-Ab=6 (5,35%)<br>-Onconeuronal=1 (0,89%) (anti-HU).		
Gozubatik-Celik G, 2017	Design:<br>Observational cross-sectional study<br><br>Objective:<br>To investigate the presence of neuronal autoantibodies in focal epilepsy with unknown cause and	Age: ≥ 18 years.<br><br>Patients with structural lesions in brain magnetic resonance imaging (MRI) such as tumor or dysplasia were excluded from the study. However, patients with focal or diffuse atrophy or nonspecific white matter	plasma:<br>-NMDAR-Ab (IF CBA)<br>-LGI1-Ab (IF CBA)<br>-CASPR2-Ab (IF CBA)<br>-GABAb-Ab (IF CBA)<br>-AMPAR-Ab (IF	-Nº of patients with positive result=2 (2,12%)<br><br>-Nº of controls with positive result=0 (0,0%)<br><br>Bi autoantibody type:	- Six patients GAD positive but without saying titers<br><br>- Semiquantitive Scale for IF from 0-4. Positive when >1.	2+

	their clinical correlates in both drug-responsive and resistant patients.<br><br>Recruitment: Prospective 12 months	hyperintensities were not excluded. In addition, none of the included patients had current findings or past medical history of any neurological conditions. Patients with systemic autoimmune disorders, febrile seizures or systemic infections were included if there was no direct temporal association between these medical conditions and the onset of seizures. Likewise, patients with mesial temporal lobe epilepsy with hippocampal sclerosis were not excluded since their seizure features and locations could not be explained by hippocampal sclerosis alone. Seizures and syndromes were diagnosed according to the International League Against Epilepsy (ILAE) Commission on Classification and Terminology 2014. All MRI studies were performed with 1.5 T scanners with thin coronal, sagittal and axial planes including T1, T2, and fluid-attenuated inversion recovery (FLAIR) images.<br><br>Number of patients included for metanalysis (Inclusion/Exclusion criteria met for our study):<br>- N=94 (HS not excluded<br><br>-Control group: n=50 healthy age and gender paired	CBA)<br>-GAD-Ab (RIA)	- NMDAR-Ab= 1 (1,06%)<br>-AMPAR-Ab=1 (1,06%)		

Iorio, 2015	Design:<br>Observational prospective<br><br>Objective:<br>the frequency of neural autoantibodies in patients with epilepsy due to unknown cause and the response to immunotherapy in patients with AED-resistant seizures were evaluated. Materials<br><br>Recruitment:<br>Prospective	Group 1 included patients with epilepsy due to unknown etiology responding to ≤2 different AEDs associated with at least one of the following conditions: psychiatric disturbances (psychosis, delirium); movement disorders (dystonia, chorea, tremor); cognitive impairment (with subacute onset); associated autoimmune diseases (e.g., lupus, Sjogren, myasthenia gravis). Group 2 included patients with AED-resistant epilepsy defined according to the definition of the International League Against Epilepsy Commission on Therapeutic Strategies. Patients with structural/metabolic epilepsy were excluded from the study. Electroencephalography and brain magnetic resonance imaging (MRI) were performed in all patients enrolled in the study<br><br>Number of patients included for metanalysis (Inclusion/Exclusion criteria met for our study):<br>N=42 with drug resistant epilepsy<br>Mean Age 36 +/-16.1<br><br>Control group:<br>Yes<br>N=75; healthy sex-age matched	Serum (CSF if aviable)<br><br>IF ICH (onconeuronals confirmed by immunoblot and surface by CBA); GAD65 by RIA<br>- Onconeuronals<br>- NMDAR<br>- AMPA<br>- GABAb<br>- LGI1<br>- CASPR2<br>- GAD65	-Nº of total patients with positive result=6 (14,28%)<br><br>By autoantibody type:<br>- GAD65=2<br>- LGI1=3<br>- NMDAR=1	Study with to different cohorts. Only second cohort included (first cohort exclusion criteria)<br><br>In methods the say about control group but any information is lacking in the rest of the article	2+

Ekizoglu E, 2014	Design:<br>Observational cross-sectional study<br><br>Objective:<br>To investigate the prevalence of these autoantibodies in patients with focal epilepsy of unknown cause and in the group having mesial temporal lobe epilepsy with hippocampal sclerosis.<br><br>Recruitment:<br>Prospective.	adult patients diagnosed with FEoUC or MTLE-HS. Seizures and syndromes were diagnosed according to the revised terminology, and concepts for organization of seizures and epilepsies of the International League Against Epilepsy (ILAE) Commission on Classification and Terminology,1 and their auras were classified according to Report of the ILAE Task Force on Classification and Terminology.10 The patients with obvious provoking factors or an apparent remote origin, such as a brain malformation or tumor, trauma, central nervous system infection with magnetic resonance imaging (MRI) evidence, or generalized epilepsy were excluded. All MRI studies were performed with 1.5 T scanners with thin coronal in addition to sagittal and axial planes including T1, T2, and fluid-attenuated inversion recovery (FLAIR) images to visualize mesial temporal regions optimally. All EEG and video-EEG monitoring records were reviewed retrospectively by the author<br><br>Number of patients included for metanalysis (Inclusion/Exclusion criteria met for our study):	Serum:<br>-NMDAR-Ab (IF CBA)<br>-LGI1-Ab (IF CBA)<br>-CASPR2-Ab (IF CBA)<br>-AMPAR-Ab (IF CBA)<br>-GLY-R (IF CBA)<br>-GAD-Ab (IPA)	-Nº of patients with positive result=11 (13,58%). With HS n=5 (19,23%)<br><br>-Nº of controls with positive result=0 (0,0%)<br><br>By autoantibody type:<br>-NMDAR-Ab=2 (2,46%)<br>-CASPR2-Ab= 4 (4,93%)<br>-GLY-R= 5 (6,17%)	Semiquantitive Scale for IF from 0-4. Positive when >1.	2++

		- n= 81 patients; 26/81 with HS, ≥ 18 years<br><br>- Control group: n=30 healthy subjects				
Brenner T, 2013	Design:<br>Observational cross-sectional study<br><br>Objective:<br>To determine the prevalence of autoantibodies in patients with stablished epilepsy and new onset epilepsy<br><br>Recruitment:<br>Prospective.	established epilepsy or new-onset epilepsy. Subjects with a history of alcohol or recreational drug abuse, suspected nonepileptic seizures, or a progressive neurologic disorder were specifically excluded. Appropriate<br><br>Number of patients included for metanalysis (Inclusion/Exclusion criteria met for our study):<br>-N=176 patients with focal epilepsy of unknown etiology. ≥ 16 years.<br><br>- Control group: n=148. Healthy subjects 30/148. Neurological patients without epilepsy 98/148. Autoimmune diseases 20/148.	Serum:<br>-NMDAR-Ab (IF CBA)<br>-LGI1-Ab (IF CBA)<br>-CASPR2-Ab (IF CBA)<br>-GLY-R (IF CBA)<br>-GAD-Ab (RIA)	-Nº of patients with positive result= 14 (7,95%)<br><br>-Nº of controls with positive result= 0 (0,0%)<br><br>By autoantibody type:<br>-NMDAR-Ab= 5 (2,84%)<br>- GLY-R= 9 (5,02%)	-A positive LGI1 result is not included in the analysis because it does not specify whether it corresponds to the group of epilepsy of unknown etiology.<br><br>-4 patients with positive GAD-Ab are not included because they did not specify a titre> 1000 U / ml.<br><br>-It is unknown whether they consider hippocampal sclerosis a structural etiology.	2+
Falip M, 2012	Design:<br>Observational cross-sectional study<br><br>Objective:<br>To describe the prevalence of GAD-Ab in patients with temporal lobe epilepsy with/without HS and to	patients with epilepsy onset beyond the age of 30 and with clinical (using seizure semiology) MRI and EEG features of TLE, whether associated or not with hippocampal sclerosis (HS). Known aetiology: 19 patients with a confirmed initial precipitating injury: nine patients had viral encephalitis or meningitis, five had personal history of febrile seizures and	Serum (CSF if aviable):<br>-GAD-65Ab (IF ICH and RIA)	-Number of patients with a positive result= 2 (8,7%)<br><br>By autoantibody type<br>-GAD-65Ab= 2 (8,7%)	-High GAD-65Ab titers considered> 1000 U / ml.	2+

	characterize the clinical-immunological profile of TLE patients with high levels of GAD-ab<br><br>Recruitment: Prospective 18 months.	five had severe head trauma. Unknown aetiology: patients with no initial precipitating injury and no<br><br>Number of patients included for metanalysis (Inclusion/Exclusion criteria met for our study):<br>-N=23 patients with temporal lobe epilepsy of unknown etiology >30 years. (HS not excluded)<br><br>- Control group: No				
Liimatainen S, 2010	Design: Observational cross-sectional study<br><br>Objective: To Study the prevalence of anti-GAD in a cohort of patients with focal and generalized idiopathic epilepsy<br><br>Recruitment: Prospective 24 months.	patients with epilepsy and recurrent seizures treated including patients with refractory focal epilepsy. Patients with dementia or high-grade brain tumor and epilepsy were excluded from the study. Focal epilepsy types were categorized into temporal lobe (TLE), frontal lobe (FLE), parietal lobe (PLE), occipital lobe (OLE), multifocal, or unknown focal epilepsies according to the International League Against Epilepsy (ILAE) guidelines (Commission, 1989), based on the seizure semi- ology, electroencephalography (EEG)/video-EEG, and etiology. IGEs were categorized into unclassified IGE, juvenile myoclonic epilepsy (JME), childhood absence epilepsy (CAE), or juvenile absence	Serum:<br>-GAD-Ab (RIA).<br>If positive, then confirmed by IHC/immunoblot and also determination in CSF	-Nº of patients with positive result= 3 (4,1%)<br><br>By autoantibody type<br>-GAD-Ab= 3 (4,1%)	-The 3 positive patients had temporal lobe epilepsy (1 with intrathecal synthesis).	2++

		epilepsy (JAE). Etiology was defined based on the brain magnetic resonance imaging (MRI), histologic analysis in some tumors, and medical history into the following categories: hippocampal sclerosis (HS) + dual pathology (HS associated with another brain lesion), cortical dysplasia (CD) (cortical dysgenesis, heterotopia, tuberosis sclerosis), other (tumor, vascular malformation, vascular lesion, trauma, other hippocampal abnormality, CNS infection, local or diffuse atrophy. specific signal change, demyelination, or non-specific gliosis) and cryptogenic. Ninety-three percent of the patients had undergone a high resolution 1.5 Tesla brain MRI with a specific epilepsy protocol. The evaluation of brain atrophy was based on an MRI examination performed on a 1.5 or 3 Tesla machine or computer tomography (CT)<br><br>Number of patients included for metanalysis (Inclusion/Exclusion criteria met for our study):<br>-n= 73 patients with focal epilepsy of unknown etiology ≥ 16 years. (HS patients excluded from unknown etiology group)<br><br>- Control group: N=200 non-Diabetic non epilepsy organ donors				

Supplementary Table S2: Excluded studies and their reason.

Study	Reason for exclusion
McGinty 2020(1)	Etiology of new-onset epilepsy no specified (even in supplementary material)
Zhang 2020(2)	Retrospective recruitment
Kuehn 2020(3)	Cohort of patients with suspected autoimmune epilepsy exclusively
Liu 2020(4)	Retrospective. Cohort of patients with suspected autoimmune encephalitis exclusively. Some patients with seizures but not epilepsy.
Bozzetti 2020(5)	Retrospective recruitment. Patients <16 years.
Tizazu 2020 (6)	Retrospective. Patients with etiology different from unknown etiology included (example, 2 patients with positive results etiology were viral encephalitis and TBI)
Zelano 2019 (7)	ILAE criteria for epilepsy not met. Epilepsy of various etiologies included.
Nóbrega-Jr 2018 (8)	Hippocampal sclerosis of different etiologies included (even MS). Not possible to extract data from patients with unknown etiology
Elisak 2018 (9)	Patients with temporal lobe epilepsy "Irrespective of MRI findings". Patients with all TLE aetiologies, except primary neuroglial tumours and brain metastasis were included.
Lv 2018 (10)	Retrospective.
Vanli-Yavuz 2016 (11)	Included patients with hippocampal sclerosis with classical risk factors included as febrile seizure, meningitis, birth trauma and head trauma as well as patients with cognitive disfunction.
Ceyhan-Dirican 2016 (12)	Included patients with drug-resistant epilepsy with hippocampal sclerosis with classical risk factors included as febrile seizure, CNS infection and birth trauma.
Lilleker 2013 (13) and 2014 (14)	Retrospective recruitment
Errichiello 2009 (15)	Patients < 16 years
McKnight 2005 (16)	Patients with drug resistant epilepsy of various etiologies (example cortical dysplasia, neoplasia, abscess etc..)
Sokol 2004 (17)	GAD titles absent
Verrotti 2003 (18)	Patients with different etiologies included
Kwan 2000 (19)	Hippocampal sclerosis etiology not specified. Cannot be assumed unknown etiology.
Peltola 2000(20)	Different etiologies included
Dambinova 1997 (21)	Epilepsy and etiologies not defined

1. McGinty RN, Handel A, Moloney T, Ramesh A, Fower A, Torzillo E, et al. Clinical features which predict neuronal surface autoantibodies in new-onset focal epilepsy: implications for immunotherapies. J Neurol Neurosurg Psychiatry. 2020 Nov;

2. Zhang W, Bu H, Li Y, Han X, He J, Jia L, et al. Development and validation of a predictive model for the diagnosis of neural antibody-mediated epilepsy/seizure in patients with new-onset seizure or established epilepsy. Seizure. 2020 Dec;83:5–12.
3. Kuehn JC, Meschede C, Helmstaedter C, Surges R, von Wrede R, Hattingen E, et al. Adult-onset temporal lobe epilepsy suspicious for autoimmune pathogenesis: Autoantibody prevalence and clinical correlates. Biagini G, editor. PLoS One. 2020 Oct 29;15(10 October):1–18.
4. Liu W-P, Wang M, Zhang C, Zhao CW, Xiao B, Zeng C. Application of the APE(2)-CHN and RITE(2)-CHN scores for autoimmune seizures and epilepsy in Chinese patients: A retrospective study. Seizure. 2020 Oct;81:63–70.
5. Bozzetti S, Rossini F, Ferrari S, Delogu R, Cantalupo G, Marchioretto F, et al. Epileptic seizures of suspected autoimmune origin: A multicentre retrospective study. J Neurol Neurosurg Psychiatry. 2020 Nov;91(11):1–9.
6. Tizazu E, Ellis CA, Reichert J, Lancaster E. Low rate of glutamic acid decarboxylase 65 (GAD-65) antibodies in chronic epilepsy. Seizure. 2020 Aug;80:38–41.
7. Zelano J, Axelsson M, Constantinescu R, Malmeström C, Kumlien E. Neuronal antibodies in adult patients with new-onset seizures: A prospective study. Brain Behav. 2019 Nov;9(11):e01442.
8. Nóbrega-Jr AW, Gregory CP, Schlindwein-Zanini R, Neves F de S, Wolf P, Walz R, et al. Mesial temporal lobe epilepsy with hippocampal sclerosis is infrequently associated with neuronal autoantibodies. Epilepsia. 2018 Sep;59(9):e152–6.
9. Elisak M, Krysl D, Hanzalova J, Volna K, Bien CG, Leypoldt F, et al. The prevalence of neural antibodies in temporal lobe epilepsy and the clinical characteristics of seropositive patients. Seizure. 2018 Dec;63:1–6.
10. Lv R-JR-JR-J, Ren H-TH-T, Guan H-ZH-Z, Cui T, Shao X-QX-Q. Seizure semiology: an important clinical clue to the diagnosis of autoimmune epilepsy. Ann Clin Transl Neurol. 2018 Feb;5(2):208–15.
11. Vanli-Yavuz EN, Erdag E, Tuzun E, Ekizoglu E, Baysal-Kirac L, Ulusoy C, et al. Neuronal autoantibodies in mesial temporal lobe epilepsy with hippocampal sclerosis. J Neurol Neurosurg Psychiatry. 2016 Jul;87(7):684–92.
12. Ceyhan Dirican A, Elibirlik S, Köksal A, Öztürk M, Altunkaynak Y, Baybaş S, et al. Evaluation of glutamic acid decarboxylase antibody levels in patients with juvenile myoclonic epilepsy and mesial temporal lobe epilepsy with hippocampal sclerosis. Noropsikiyatri Ars. 2016 Sep;53(3):253–6.
13. Lilleker JB, Jones MS, Mohanraj R. VGKC complex antibodies in epilepsy: diagnostic yield and therapeutic implications. Seizure. 2013 Nov;22(9):776–9.
14. Lilleker JB, Biswas V, Mohanraj R. Glutamic acid decarboxylase (GAD) antibodies in epilepsy: diagnostic yield and therapeutic implications. Seizure. 2014 Sep;23(8):598–602.
15. Errichiello L, Perruolo G, Pascarella A, Formisano P, Minetti C, Striano S, et al. Autoantibodies to glutamic acid decarboxylase (GAD) in focal and generalized epilepsy: A study on 233 patients. J Neuroimmunol. 2009 Jun;211(1–2):120–3.
16. McKnight K, Jiang Y, Hart Y, Cavey A, Wroe S, Blank M, et al. Serum antibodies in epilepsy and seizure-associated disorders. Neurology. 2005 Dec;65(11):1730–6.
17. Sokol DK, McIntyre JA, Wagenknecht DR, Dropcho EJ, Patel H, Salanova V, et al. Antiphospholipid and glutamic acid decarboxylase antibodies in patients with focal epilepsy. Neurology. 2004 Feb;62(3):517–8.
18. Verrotti A, Greco R, Altobelli E, Latini G, Morgese G, Chiarelli F. Anticardiolipin, glutamic acid decarboxylase, and antinuclear antibodies in epileptic patients. Clin Exp Med. 2003 May;3(1):32–6.
19. Kwan P, Sills GJ, Kelly K, Butler E, Brodie MJ. Glutamic acid decarboxylase autoantibodies in controlled and uncontrolled epilepsy: a pilot study. Epilepsy Res. 2000 Dec;42(2–3):191–5.
20. Peltola J, Kulmala P, Isojärvi J, Saiz A, Latvala K, Palmio J, et al. Autoantibodies to glutamic acid decarboxylase in patients with therapy-resistant epilepsy. Neurology. 2000 Jul;55(1):46–50.
21. Dambinova SA, Izykenova GA, Burov S V, Grigorenko E V, Gromov SA. The presence of autoantibodies to N-terminus domain of GluR1 subunit of AMPA receptor in the blood serum of patients with epilepsy. J Neurol Sci. 1997 Nov;152(1):93–7.
